# Health enhancing physical activity in patients with hip or knee osteoarthritis - an observational intervention study

**DOI:** 10.1186/s12891-017-1394-7

**Published:** 2017-01-25

**Authors:** Anna Ernstgård, MirNabi PirouziFard, Carina A. Thorstensson

**Affiliations:** 1 Linneaus University, Kalmar, Sweden; 2 Capio Artro Clinic AB, Box 5606, Stockholm, SE-114 86 Sweden; 3 Centre of Registers Västra Götaland, Gothenburg, Sweden; 4 Department of Clinical Neuroscience and Rehabilitation, Institute of Neuroscience and Physiology, The Sahlgrenska Academy, University of Gothenburg, Gothenburg, Sweden; 5 BOA-registry, Centre of Registers Västra Götaland, Gothenburg, Sweden

**Keywords:** Exercise, Osteoarthritis, Patient education, Registry

## Abstract

**Background:**

Osteoarthritis is one of the leading causes of inactivity worldwide. The recommended level of health enhancing physical activity (HEPA) is at least 150 min of moderate intensity physical activity per week. The purpose of this study was to explore how the proportion of patients, who reached the recommended level of HEPA, changed following a supported osteoarthritis self-management programme in primary care, and to explore how reaching the level of HEPA was influenced by body mass index (BMI), gender, age and comorbidity.

**Methods:**

An observational study was conducted using data from a National Quality Registry in which 6810 patients in primary care with clinically verified hip or knee osteoarthritis with complete data at baseline, 3 and 12 months follow-up before December 31^st^ 2013 were included. HEPA was defined as self-reported physical activity of at least moderate intensity either a) at least 30 min per day on four days or more per week, or b) at least 150 min per week. HEPA was assessed at baseline, and again at 3 and 12 months follow-up. Cochran’s Q test was used to determine change in physical activity over time. The association between reaching the level of HEPA and time, age, BMI, gender, and Charnley classification was investigated using the generalized estimation equation (GEE) model.

**Results:**

The proportion of patients who reached the level of HEPA increased by 345 patients, from 77 to 82%, from baseline to 3 months follow-up. At 12 months, the proportion of patients who reached the level of HEPA decreased to 76%. Not reaching the level of HEPA was associated with overweight, obesity, male gender and Charnley category C, i.e. osteoarthritis in multiple joint sites (hip and knee), or presence of any other disease that affects walking ability.

**Conclusions:**

Following the supported osteoarthritis self-management programme there was a significant increase in the proportion of patients who reached the recommended level of HEPA after 3 months. Improvements were lost after 12 months. To increase physical activity and reach long-lasting changes in levels of physical activity, more follow-up sessions might be needed.

## Background

Osteoarthritis is the most common joint disorder [1]. According to the World Health Organisation (WHO) 9.6% of men and 18% of women aged over 60 have osteoarthritis worldwide [2, 3]. The disease often results in pain and impaired mobility and it is one of the leading causes of disability around the world [2–4].

The core treatments of osteoarthritis consist of patient education, exercise and weight control [5–7]. These basic treatments may be supplemented by pharmacological treatment and/or orthopaedic devices. Joint replacement should only be considered if the above mentioned treatments are non-sufficient [5].

In patients with osteoarthritis, physical inactivity is a predictor of increased symptoms and poor general health [8–10]. In spite of this, physical inactivity is common among patients with osteoarthritis [11]. Presence of comorbidity together with osteoarthritis is associated with limitations in physical activity [12–14]. Overweight and obesity may also contribute to lack of physical activity [15, 16]. Further, the level of physical activity usually decreases with older age [17, 18] and varies greatly between countries. Using self-reported physical activity for adults, meeting the recommendations (as defined by individual studies) range from 2% in Taiwanese and Saudi Arabian women to 81% in women in Denmark. In men, the range goes from 4% in Brazil to 77% in Swedish men [19]. In a recent study, based on accelerometer monitoring, 12.9% of men and 7.7% of women in the United States met physical activity recommendations [11]. This can be compared to data from the 2002 National Health Interview Survey in the United States where 38% without baseline osteoarthritis met the current recommendations [20]. Physical inactivity increases the risk of many chronic conditions, such as cardiovascular diseases, type 2 diabetes, obesity, colon cancer, breast cancer, dementia and depression [10, 21, 22]. The WHO recommendation proposes physical activity for at least 150 min per week at moderate intensity, or high-intensity physical activity for at least 75 min per week, in order to gain health benefits. Moderate and high-intensity physical activities can be combined to achieve the recommended amount of physical activity [10]. These recommendations were first published in 2007 [23] and further refined in 2010 [10]. The earlier recommendation from 1995 was to accumulate at least 30 min of moderate physical activity on most days of the week [24].

Efforts to increase levels of physical activity in patients with osteoarthritis are important to increase health-related quality of life as well as to decrease social costs related to sick leave and healthcare consumption [2, 9]. Self-management programmes have been created to help this group in a cost-efficient way. A physiotherapist-delivered combined psychological and exercise intervention was found to be beneficial for patients with osteoarthritis. It could however not demonstrate cost-efficiency [25]. Evaluation of an arthritis self-management programme showed that long-term maintenance of self-efficacy, psychological well-being and self-management may be possible following the programme [26]. Significant improvement in physical activity in the short term was found in a review article with meta-analysis on the physical activity level following self-management programmes for lower limb osteoarthritis. However, the effectiveness of the interventions declined after 12 months [27].

The Swedish national programme *Better management of patients with osteoarthritis* (BOA) was initiated in 2008 to offer all patients with hip and knee osteoarthritis information and individually adapted training in accordance with current national and international treatment guidelines for osteoarthritis. The BOA has three branches: education of patients, training of healthcare professionals, and the National Quality Registry, the BOA-registry [28, 29]. Patient education consists of an evidence-based supported osteoarthritis self-management programme and has formed part of primary care in Sweden since 2010. It is delivered by several hundred specifically trained physiotherapists at hundreds of primary care centres all around Sweden. The purpose of this study was to explore how the proportion of patients who reached the recommended level of health enhancing physical activity (HEPA) changed following a supported osteoarthritis self-management programme in primary care, and to explore how reaching the level of HEPA was influenced by body mass index (BMI), gender, age and comorbidity.

## Methods

**Fig. 1:**
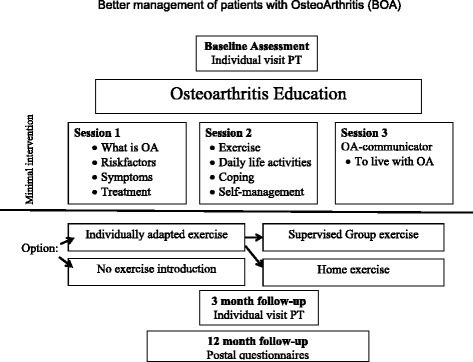
Disposition of the supported osteoarthritis self-management programme

The study was conducted as an observational study using data from a National Quality Registry, the BOA-registry, comprising patients with hip or knee osteoarthritis who participated in a supported self-management programme in primary care in all regions of Sweden. The programme has been described in detail elsewhere [28]. In brief it consists of a minimum of two theoretical group sessions led by a physiotherapist. During these sessions information about osteoarthritis and its treatment is presented, with special focus on self-management, including physical activity. The theoretical sessions aim, among other things, to explain the mechanisms behind the possible benefits of specific exercises and to increase the patients’ motivation to exercise and become physically active. Individually adapted and supervised exercise is optional. The individual exercise programme is based on the patient’s specific needs and goals, and presented and tried out during a one-to-one session. Patients can choose to perform exercises on their own, or to attend physiotherapist-supervised exercise classes twice a week for six weeks along with others from the programme, but using their individual programme. The physiotherapist provides support, advice and individual adjustments when needed. Patient-reported outcomes are used at baseline, 3 and 12 months follow-up (Fig. 1) [28, 29]. These data are collected in a National Quality Registry, the BOA-registry [29].

### Study population

Data on patients with clinically verified hip or knee osteoarthritis, consecutively included between 2008 and 2012 and with complete data from 3 and 12 months follow-up, were compiled and analysed. Patients were eligible for the intervention if they presented with non-traumatic pain sufficient to seek primary care and attributed by a clinician to the hip or knee. In accordance with the National Board of Health and Welfare guidelines for osteoarthritis, published in May 2012 [30], hip or knee osteoarthritis was determined through the patient’s medical history, typical symptoms and a clinical examination. According to these guidelines, radiographic examination is only to be used in uncertain cases, or when a surgical intervention is planned. Simultaneous osteoarthritis of other joints did not preclude participation. Patients with inflammatory joint disease, serious illness, sequel hip-fracture, chronic widespread pain or difficulties in understanding the Swedish language were excluded from the registry. Patients went through the programme in primary care setting in both countryside and metropolitan areas in all regions of Sweden. Areas with different socioeconomic status were represented [29].

### Outcomes

Until September 1^st^ 2012, HEPA in the BOA-registry was defined as “at least 30 min per day on most days”. “Most days” in the present study was recognised as four days or more per week. Patients answered the question “How many days per week do you usually accumulate at least 30 min of physical activity?” by choosing one answer from 0 days to 7 days. By September 1^st^ 2012, the new definition of HEPA (150 min of physical activity per week) was introduced, using two questions recommended by the National Board of Health and Welfare. Patients included in the intervention after this date answered the following questions: “How much time do you spend, during an ordinary week, on physical activity, e.g. walking, cycling or gardening?” and “How much time do you spend, during an ordinary week, on physical exercise which makes you breathless, such as e.g. running, aerobics or ball sports?” Each question has several alternative answers regarding minutes spent in physical activity: “no time”, “less then 30 min”, “30–60 min”, “60–90 min”, “90–150 min”, “150–300 min” and “more than 300 min”, and for physical exercise: “no time”, “less then 30 min”, “30–60 min”, “60–90 min”, “90–120 min” and “more than 120 min”. The median value of each interval was used for calculation for the individual. For this definition of HEPA, the total number of activity minutes was calculated by doubling the number of minutes of physical exercise and then adding the minutes of physical activity [29, 31, 32]. In the present study, patients reporting either physical activity of a moderate intensity on four days per week or more at least 30 min per day, or at least 150 min of physical activity per week, were classified as reaching the level of HEPA. Level of physical activity was then dichotomised, as reaching the level of HEPA – yes or no – at each time point. The changes in level of physical activity between baseline, 3 and 12 months were investigated.

### Covariates

Self-reported age, gender, BMI and comorbidity according to the Charnley classification were extracted from the register. The Charnley classification categorises patients into one of three groups: A - one joint with osteoarthritis (unilateral knee or hip); B - bilateral osteoarthritis (both knees or both hips); C - osteoarthritis in multiple joint sites (hip and knee), or presence of any other disease that affects walking ability [33].

BMI was classified according to WHO: < 18.5 kg/m^2^ was classified as underweight, 18.5–25 kg/m^2^ as normal weight, ≥ 25 kg/m^2^ was classified as overweight, and a BMI of 30 kg/m^2^ or more was classified as obesity [34]. Due to low numbers in the underweight category, underweight and normal weight patients were merged into one category.

Four age groups of similar size were created: younger working age (22–54 years), older working age (55–64 years), younger retirees (65–74 years) and older retirees (75+ years).

### Statistical analysis

Cochran’s Q test was used to determine differences in physical activity between baseline and follow-up at 3 and 12 months. A *p*-value of <0.05 was considered to be statistically significant.

A generalised estimation equation (GEE) model was used to investigate the differences in characteristics between the patients who reached the recommended level of HEPA and those who did not. The associations were investigated between reaching the level of HEPA and the factors: time, age, BMI, gender, and Charnley classification. The time factor describes the association between reaching the level of HEPA and the different assessment times (baseline, 3 and 12 months). The GEE model allowed the patient to contribute to the analyses three times. In our analysis the dependent (response) variable was dichotomous (reaching the level of HEPA, yes or no) and the GEE model was based on logistic regression. The effects of the independent variables time, age, BMI, gender, and Charnley classification on the odds of not reaching the level of HEPA were estimated. Crude odds ratios, adjusted odds ratios and 95% confidence intervals were calculated.

## Results

**Fig. 2:**
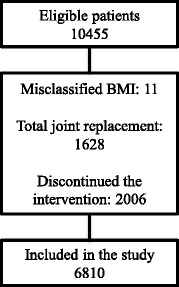
Flow chart over study participants

For the present study 10455 patients were eligible, and 6810 (65%) fulfilled the inclusion criteria (Fig. 2). Eleven patients had irrational BMI (extreme outliers), 1628 (11%) patients had hip or knee replacement surgery before one year follow up, and 2006 (14%) patients chose to discontinue the intervention. The patients included comprised 73% women, had a mean age of 65 (SD 9) years, and a mean BMI of 28 (SD 5) kg/m^2^ (height or weight were missing for 1.6% of the patients, thus BMI could not be calculated). More patients reported larger problems with knee pain compared to hip pain (73 vs. 27%). The classification according to Charnley category A, B and C resulted in 35% of the patients in category A, 31% in category B and 33% in category C (0.5% of the patients did not report whether they had uni- or bilateral problems, leading to missing values of Charnley classification) (Table 1).

**Table 1:** Descriptives of the study population (*N* = 6810), predictors, crude and adjusted odds ratios (OR) with 95% confidence intervals (95% CI)

Predictor	Mean (SD)	Group	%	Crude OR (95% CI)	Adjusted OR (95% CI)
Time	n.a.	0 month	n.a.	1.00	1.00
		3 month	n.a	0.83 (0.77–0.88)	0.78 (0.72–0.84)
		12 month	n.a	1.07 (1.00–1.15)	1.10 (1.02–1.19)
Age	65 (9)	22–54 years		1.00	1.00
		55–64 years		0.75 (0.65–0.85)	0.74 (0.64–0.85)
		65–74 years		0.50 (0.44–0.57)	0.47 (0.42–0.54)
		75–100 year		0.78 (0.67–0.91)	0.64 (0.55–0.75)
Charnley category^a^	n.a.	A	35	1.00	1.00
		B	31	1.05 (0.94–1.17)	1.00 (0.90–1.11)
		C	33	1.38 (1.25–1.53)	1.15 (1.04–1.28)
BMI	28 (5)	Normal weight		1.00	1.00
		Overweight		1.39 (1.25–1.56)	1.27 (1.14–1.42
		Obesity		2.36 (2.10–2.65)	1.93 (1.71–2.17)
Gender	n.a.	Man	27	1.00	1.00
		Woman	73	0.80 (0.73–0.88)	0.69 (0.62–0.75)

*n.a.*not applicable

^a^Charnley category A: One joint with osteoarthritis (knee or hip). B: Bilateral osteoarthritis (both knees or both hips) C: Osteoarthritis in multiple joint sites (hip and knee), or presence of any other disease that affects walking ability

**Fig. 3:**
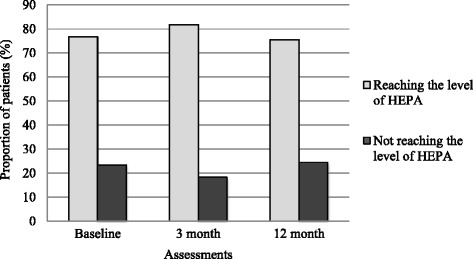
Distribution between physically active and inactive at baseline and at follow-up sessions. HEPA (health enhancing physical activity) was defined as self-reported physical activity of at least moderate intensity either a) at least 30 min per day on 4 days or more per week, or b) at least 150 min per week

The proportion of patients who reached the level of HEPA increased from 77% at baseline to 82% at the follow-up after 3 months (*p* < 0.001). At follow-up after 12 months the proportion decreased to 76% (Fig. 3).

**Fig. 4:**
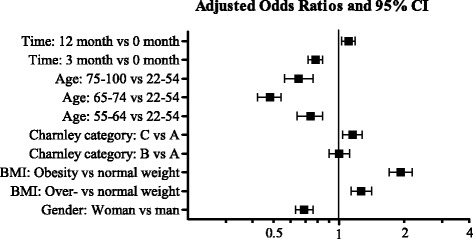
The results of the GEE analysis, the box denote odds ratio (OR) with 95% confidence interval (CI). An OR greater than 1 means increased risk not to reach the level of health enhancing physical activity, while an OR below 1 means reduced risk

The results of the GEE analysis showed that not reaching the recommended level of HEPA was associated with overweight, obesity and male gender. Patients with one joint affected by osteoarthritis (Charnley category A) reached the recommended level of HEPA to a greater extent compared to patients with both hip and knee problems or walking disabilities for other reasons (Charnley category C). Fewer patients of younger age (22–54 years) reached the recommended level of HEPA compared with patients in the other age groups. The “time” result showed that a larger proportion of the patients reached the level of HEPA at the 3 months follow-up than at baseline. This result was consistent with the result of the Cochran’s Q test (Table 1, Fig. 4).

## Discussion

A greater proportion of patients reached the recommended level of HEPA after completion of the supported osteoarthritis self-management programme. However, overall this increase was lost at follow-up after one year. The result confirms the conclusion of a review article with meta-analysis on physical activity level following self-management programmes for lower limb osteoarthritis [27] and the short-term result confirms previous research on a similar intervention [35]. To achieve long lasting increased physical activity through an intervention it is important to individualise the activity according to the patient’s preferences, resources and goals [6]. The patients in the supported osteoarthritis self-management programme form a heterogeneous group. Individuals need different levels of support to make lifestyle changes [36]. Many patients with osteoarthritis are familiar with the benefits of physical activity for health, but have doubts and concerns about specific exercises and physical activity as a form of treatment for osteoarthritis [36]. The results indicate that patients with overweight and obesity, who are more likely to not reach the recommended level of HEPA, might need extra support. This is consistent with other studies [15, 16, 37]. The cause and consequence relationship is hard to determine. Obese individuals with osteoarthritis have a higher prevalence of comorbidity [38]. The presence of comorbidity and overweight may contribute to reduced physical activity, but decline in physical activity and obesity may also cause the occurrence of other illnesses. Osteoarthritis in combination with comorbidity is associated with more pain [12–14, 39] and further limitations in physical activity [12–14]. This was confirmed in our study, where a larger proportion of patients with both hip and knee problems, or walking disabilities for other reasons (Charnley category C) did not reach the recommended level of HEPA. In the population studied, 60% of the patients with hip osteoarthritis and 40% of patients with knee osteoarthritis were classified as Charnley C [40]. The supported osteoarthritis self-management programme may only have limited possibilities to positively affect other disabilities causing walking impairment.

Variations between therapists in fidelity and practice, as well as patients’ cognition and mood may influence the way patients perceive the information provided, and thus the treatment outcome [41]. Pain is a common symptom in osteoarthritis. Pain can both decrease and increase over time and may also affect the level of physical activity, as well as changes in levels of physical activity may affect pain intensity. During the intervention, ways to overcome this barrier are presented to the patients, and ways of pain management during activity are discussed. In this study we have not investigated how pain possibly influenced our results. Today, the best exercise regimen for this group of patients is not known [6, 42], but to achieve long-lasting behavioural changes some contact or follow-up might be of importance [6]. To change health behaviour and to maintain a healthy lifestyle takes a long time and continuous commitment [43]. It is possible that more follow-up or booster sessions could improve the long-term results, and enhance physical activity.

The dataset was extracted from a National Quality Registry, continuously and consecutively enrolling patients participating in the supported osteoarthritis self-management programme in primary care. Thus, the current study represent everyday multi-site clinical primary care practice in Sweden, where factors related to the therapist performance, patient perception and clinical practice are hard to assess, but might influence outcome. The supported osteoarthritis self-management programme includes optional supervised rehabilitation group training for six weeks. Patients in the group use individual programmes, but exercise together. The programme is tried out on an individual basis by the physiotherapist during an optional one-to-one session prior to the six-week period. During this individual session, home exercises are introduced to become a part of the patients’ everyday life. Approximately 80% of the patients opt for the individual programme, and 60% take part in the supervised rehabilitation training [40]. Reasons for not attending could for example be geographical distance, or lack of time. Not attending does not necessarily mean that patients are not performing their individually adapted exercises. In addition, not opting for an individual programme could mean that the patient is not motivated at this time, but also that they already have a programme or physical activity that works well. In the present study we have not explored how compliance to the different parts of the intervention influences the level of HEPA. A limitation of the study is the lack of information about whether or not they were exercising on their own.

A higher proportion of women reached the level of HEPA, and patients in the older age groups were more likely to reach the recommended level of HEPA compared to individuals aged between 22 and 54 years. It is possible that older patients have more time to spend on exercise and leisure activities than younger patients, building careers and families. Another possible explanation could be that older patients may have more frequent symptoms such as pain and disability, reminding them of the need for structured exercise. Gender-related level of physical activity varies between countries. In many parts of the world women are less physically active than men [11, 19, 44, 45]. In Sweden, however, the level of physical activity among men and women is more equal. Swedish women are also in general more physically active than women in the United States [44]. This, together with the high proportion of women included in this study, may further contribute to a high proportion of patients reaching the level of HEPA already before the intervention, compared to previously published studies from other countries.

Statistics from WHO 2014 show that approximately two-thirds of the adults in Sweden reached the recommended level of HEPA [46]. The greater proportion of patients reaching the level of HEPA in the present study (77%) may partly be explained by the fact that patients with inflammatory joint diseases, serious illnesses, sequel hip-fracture or chronic wide spread pain are excluded from the registry. Another possible explanation could be the use of self-reported physical activity. The level of physical activity is difficult to measure. Current methods used include accelerometers, pedometers, training logs and different types of patient-reported outcomes. A patient-reported outcome may increase the risk of recall bias or over- or underestimation of physical activity, however it is feasible to use for large, population-based studies. More studies comparing validity between objective methods, such as accelerometers, and patient-reported outcomes are needed. It is not possible to determine how self-report might have influenced the results of this study. However, all results are based on paired analyses, and the change is calculated for every patient, probably reporting similar over- or underestimation at all time-points. Some patients may have included the supervised exercise sessions in their responses to the follow-up questions on physical activity and exercise. However, the follow-up visit was scheduled after completion of the exercise sessions, and the questions concerns an “ordinary week”. Further, this study aim to observe the results of daily practice on a national basis, and accelerometers are not available at all primary care centres, or regularly used in clinical practice. A third explanation might be related to the fact that most patients in the present study responded to questions assessing the older version of HEPA, using a more ‘generous’ limit [24] compared to the updated classification [10, 23]. The older recommendation, to be physically active at least 30 min on ‘most days of the week’, was recognised as four days or more per week, or minimum 120 min. Thus, more individuals might be classified as sufficiently physically active compared to the new recommendation of 150 min. Two different methods of calculating HEPA had to be used in the current study, based on the change of questionnaires used in the registry over time. However every patient responded to the same questionnaire at all time points. A fourth possible explanation could be that only patients who completed the 3- and 12-month follow-ups were included, and data on the level of physical activity among dropouts are lacking, which may bias the interpretation of the results. Patients completing the supported osteoarthritis self-management programme may already before be more motivated to take active engagement in their health, including physical activity.

Most studies on the impact of physical activity interventions are designed as RCTs [25, 26, 35, 47, 48]. The current observational study design could not determine the true effect of the evidence-based supported self-management programme, thus we do not know the natural variation of HEPA over time in this population. However, data in the National Quality Registry used for the present study represent clinical reality, increasing the representativity and generalisability of the results compared to randomised controlled trials (RCTs), which often use narrow inclusion criteria and less flexible programmes. A population of this magnitude, with national distribution, is not feasible to study using an RCT design. Studies on how evidence-based interventions work in reality are rare.

## Conclusions

Following the supported osteoarthritis self-management programme there was a significant increase in the proportion of patients who reached the recommended level of HEPA after 3 months. Physiotherapists may pay extra attention to patients at higher risk of not reaching the recommended level of HEPA. Factors of importance to higher risk are overweight or obesity, younger age, male gender and comorbid impairments. To achieve long-lasting changes in levels of physical activity, follow-up or booster sessions might be needed.

## Abbreviations

BMI: Body mass index
BOA: Better management of patients with osteoarthritis
GEE: Generalised estimation equation
HEPA: Health enhancing physical activity
RCT: Randomised controlled trial
SD: Standard deviation
WHO: World Health Organisation
